# Communications Through Contemporary Tools of Information and Communication Technology: Cross-sectional Study Evaluating Health Among Separated Family Members

**DOI:** 10.2196/34949

**Published:** 2022-08-03

**Authors:** Mariko Nishikitani, Mie Ariyoshi, Yasunobu Nohara, Junko Umihara

**Affiliations:** 1 Medical Information Center Kyushu University Hospital Fukuoka Japan; 2 Faculty of Health and Welfare Human Services St Catherine University Matsuyama Japan; 3 Faculty of Advanced Science and Technology Kumamoto University Kumamoto Japan; 4 Department of Career College Showa Women's University Tokyo Japan

**Keywords:** family relations, interpersonal communication, internet use, smartphone, home environment, psychosocial functioning

## Abstract

**Background:**

The number of single-living workers separated from their spouses and families has been increasing due to the need to create a balance between life and work. Workers are assigned everywhere in globalized workplaces while also caring for their family members in the context of Japan’s aging society. At the same time, the mental and health status of persons living separately from their families is a matter of concern. The development of interpersonal communication means using information and communications technology (ICT) tools and the internet is remarkable, enabling simultaneous 2-way communication across distances and national borders. The easy accessibility to simultaneous communication is expected to improve the psychosocial status of isolated family members.

**Objective:**

This study aims to clarify the health benefits of ICT by using a psychosocial health assessment, the characteristics of ICT tools, and the frequency of communication among the workers and their families who live separately.

**Methods:**

This was a cross-sectional study planned and conducted in Japan. Study participants, including adults who live separately from other family members or have separately living family members due to work, were recruited to answer a web response survey about ICT usage status, health status, and life and society evaluation. This study recruited 73 participants divided into 2 groups by their communication tools and frequencies, and their separated life, health, and psychosocial status were statistically compared.

**Results:**

Among the 73 study participants, 15 were categorized in the high communication–skilled (HCS) group that used both types of ICT tools to communicate frequently: “live,” such as video chat and voice call, and “nonlive,” such as SMS text message service and email. A simple comparison between the HCS and reference groups showed significant differences in the cohesion with the neighborhood (*P*=.03), perceived social position (*P*=.01), and happiness (*P*<.001); however, there were no significant differences in the health (psychological distress, *P*=.08; self-rated health, *P*=.07), lifestyle (drinking, *P*>.99; current smoking, *P*=.37), and dyadic trust in family members living separately (*P*=.80). Further, in a multivariate regression analysis adjusted for confounding factors, such as educational history, age, gender, and job status, poor subjective health showed a prevalence odds ratio of less than 1 (OR 0.17, 95% CI 0.03-1.02). The HCS group showed significant positive relationships in the cohesion score with the neighborhood (*P*=.01; β=2.40, 95% CI 0.56-4.24), perceived social position (*P*=.03; β=1.17, 95% CI 0.11-2.23), and happiness score (*P*=.002; β=1.46, 95% CI 0.58-2.34) in the same multivariate regression models.

**Conclusions:**

This study suggested that people who frequently communicate with separated family members by taking advantage of various ICT tools can maintain a better mental state and better social relations among those who live alone and are separated from their families.

## Introduction

In Japanese working households, couples may have a period of living separately due to raising children or caring for parents at the opportunity of a job transfer of a family member. Most Japanese workers are employees, accounting for almost 89% of the workforce [1]. Given that the Japanese company organization adopts the membership system [2], there are many cases where they follow transfer orders by their company until they reach retirement age under lifetime employment [3]. Because Japanese companies have domestic and overseas branches and departments, employees usually experience several different workplaces every few years unless they change companies. Employees belong to the company’s membership system; even if their company orders them to change their workplace, it is rare for them to leave their company because of that system. Therefore, if there are family circumstances, such as a child attending a competitive elite school or parents needing care, when workers are ordered to transfer, only the worker will leave and move to a different workplace alone. In Japan, where the birthrate is declining and the population is aging, children’s education is an important concern for families [4]. At the same time, caring for aged parents is often managed by some family members with less manpower [5]. In addition, as the working-age population is relatively small, companies have to allocate a small number of employees to various places and suitable positions. The human resource department of Japanese companies usually transfers their employees from one job to another or from one branch to other branches rather than hiring new workers to allocate necessary jobs or branch. Because they are taking the membership employment system, hiring workers means to approve the person as a member. Therefore, such an approval process is fateful and serious, and the human resource department hesitate to make decisions easily. From the high economic growth era until today, many Japanese company organizations seem not to change the membership employment system [2].

The number of single-living workers separated from their spouses and families is increasing. Although it is difficult to determine the accurate number, it has been partially figured out by the government, for example, through the Comprehensive Survey of Living Conditions and the National Census. For instance, available information aggregates male spouses who are single households as single job-transferred workers based on these national statistics [6]. According to this information, there were 750,000 single-living married men in 2015 [6], equivalent to 2.4% of the households with couples. In addition, since 1997, the Ministry of Internal Affairs and Communications has begun reporting the number of female workers living alone, although they have spouses in the Employment Status Survey. The number has also increased for women from 0.5% of female workers in 1997 to 1.2% in 2017 [7]. Thus, the percentage of single job-transferred workers for both men and women is not high in all households or workers, but it has steadily increased in the past 30 years.

The mental and health status of solitary members living apart from other family members has been a concern according to the increasing number of single job-transferred workers. Several studies on the health (in particular, mental health centered on psychological stress) of single job-transferred workers and their families were published between the 1980s and 2000s. In some studies, the health status of single job-transferred workers did not necessarily deteriorate because the age at the time of transfer, personal qualities (whether or not the transfer is positively considered), and significance of the transfer (whether or not it involves promotion) affected the situation. Therefore, the worsening effect of single job transfer on mental health did not necessarily occur in all cases [8,9]. In addition, a survey was conducted on couples in which one of them was assigned to work alone. An unpredictable life, such as not making a life plan due to an unknown assignment period, was a stress factor, although it depended on the spouses’ age and the length of the assignment [8].

Recently, physical health has been also evaluated for single job-transferred workers compared with workers living with their families. With respect to their lifestyle habits, increased smoking and frequency of drinking were higher among those assigned to work alone, and many of them did not eat breakfast [10,11]. Studies have concluded that these were due to stress. The study also reported several symptoms, such as headaches, gastrointestinal disorders, and colds, among single job-transferred workers [10]. In addition, they had a higher prevalence of mental stress, such as irritation, anxiety, and depressive mood, than workers living with family [11]. Comparing the results of health checkups of single job-transferred workers with those of workers living with their families, cholesterol levels and other values were worse for the former [11].

Workers living alone and separated from their families tend to consult with their families about health problems rather than with their close colleagues and medical staff at the working place [10]. It is suggested that communication with their families is important for maintaining their health even when they are isolated physically from them. However, few studies have focused on the communication of single job-transferred workers. Therefore, it is meaningful to evaluate the state of communication as a factor that leads to a better state of life and health of an isolated person living away from the family.

The means of communication available to families living separately have increased dramatically since the 1990s when the official number was first estimated. The development of interpersonal communication means using information and communications technology (**ICT)** tools is remarkable. In particular, the free call service using the internet network enables simultaneous 2-way communication across distances and national borders. When video functions are added, it enables visual communication and sharing of information by nonverbal transactions. Messenger apps in smartphones, such as WhatsApp, Facebook Messenger, and WeChat, are popular and each has more than 1 billion active users [12]; additionally, these apps have regional characters. In East Asia, LINE is popular in Japan, Kakao Talk is famous in South Korea, and WeChat is used the most in China [13]. These free calling apps over the internet have hundreds of millions of users worldwide and contribute as a communication tool.

Under the background of developing ICT tools, communication means in Japan has also changed. The number of general users of free calling apps, a recent method, is increasing rapidly [14]. According to the Ministry of Internal Affairs and Communications’ Communication Usage Trend Survey, the number of communications and communication hours via conventional telephones have decreased in recent years. By contrast, the number of email and social networking service (SNS) users has become more than half. It is reported that 3.8 billion people not only in Japan but also worldwide are using some kind of SNS tool today [15].

Considering communication to build interpersonal relationships, it can be said that the association between social support and health status has been known for a long time [16]. The number of people to talk to daily has been used to determine the effects of social support. The greater the number of individuals with whom one communicates, including those met face-to-face and those who communicate by email, telephone, and SNS, the higher one’s self-reported health [17]. In addition, sharing life-related information among family members through various means increases the family’s well-being [18]. In the case of spouses, increased sharing of information reduces the incidence of mental disorders in families living apart and improves their mental health [19]. It also positively affects family relationships based on affection and growth [20]. Regarding the level of sharing information, these studies evaluated the frequency of communication between families using internet-based applications (eg, Viber, Imo, and Facebook) and the number of messages sent by instant messaging as an index.

The types of ICT tools used for individual communication have been influenced by socioeconomic factors [18,21]. Nowadays, these tools are regarded as a means for maintaining and promoting health. Effective use of the internet is observed in older adults of higher socioeconomic status and in those who reported less depressive and anxiety symptoms [22]. In addition, increased access behavior to health information has been observed, as internet users are not in the lowest socioeconomic level [23]. In Japan, trials have been conducted by simultaneously sharing photographs [24] and using telemonitoring systems of television’s operating state [25] to ensure a secure and safe feeling among family members living separately. However, these are case reports of experiments among a few families, and psychological health has not been examined. In Japan, where the number of single job-transferred workers is increasing, the relationship between the physical and mental health of separated couples and their families and the usage and frequency of communication tools widely employed today is expected to be examined fully.

Currently, while numerous people are benefiting from ICT to obtain psychological support from remote family and friends, the health of those who live away from their families has not been fully evaluated in this context. Therefore, this study investigated the association between the communication via ICT and the emotional advantages among couples and families who temporarily choose to live separately because of work. This study aimed to clarify the health benefits of ICT by conducting a psychosocial health assessment as well as evaluating the characteristics of ICT tools and the frequency of communication.

## Methods

### Study Design

This was a cross-sectional study planned and conducted in Japan. Study participants were adults who live separately from families or have separately living family members due to work. Recruitment was conducted for 5 months from November 2019 to March 2020. The survey asked about ICT usage status, health status, and life and society. All answers were collected by the web response system and analyzed statistically.

### Participants

The researchers approached their acquaintances to introduce the survey to their family, friends, and colleagues. Most acquaintances were also researchers, specialists, and businessmen working in universities, research institutes, and companies with many branches located both domestically and abroad. They had experienced living alone remotely from their family and were expected to know those temporarily separated for work reasons or who had such families. As the reason for living separately, work and family care were included but not for divorce, family troubles, or other reasons. University students were excluded because many of them were usually not expected to be responsible for the life of the rest of their family members, although many of them lived alone apart from their families.

Participants were recruited by the snowball sampling method. First, by meeting directly, sending email, or calling over the telephone, the researchers asked them to be the first introducer. The researchers then asked them to send a recruitment message to the mailing list of the Young Scientist Group of the Japan Epidemiological Association. If the potential participant accepted to be an introducer, the researchers asked him/her to introduce them to 1-3 acquaintances who met the inclusion criteria and send an email of the survey site link, a token key, and the research explanatory material. If a reader of the mailing list wanted to participate in the survey and was confirmed that he/she met the criteria of living separately, the researchers directly sent a similar email with the information of a link, a token, and a research explanatory material. If the first applicants accepted the research conditions and participation in the survey, they anonymously started answering the questionnaire using the token key received. Once they finished answering the questions, a new token key and a message appeared, which asked them to send the token key to the family living separately from the first participants to allow them to participate in the survey. Finally, 73 participants were chosen to have given valid responses, and it included 12 family pairs living separately.

### Survey Variables

The survey was conducted using a free and open-source online statistical survey web app, LimeSurvey [26], and responses were collected using an anonymous self-completed survey form. Separated family pairs were identified by the tokens distributed with the survey guide. Regarding the ICT usage status, the following items were suggested to understand the types of communication tools often used: “phone calls by cellphone and telephone,” “online free calls,” “video chat using the Internet,” “short text message on cellphones,” “group text chats like LINE, FB messenger, etc.,” and “e-mail” as well as the frequency of communication with each tool from the following options: “never use,” “once a month, “few times a month,” “once a week,” “two or three times a week,” “four or five times a week,” and “almost every day.” The detailed questionnaire is presented in Multimedia Appendix 1. In addition to the basic attributes, the researchers asked about their separated living status, such as the relationship status for participants, period of separation, time and cost required to meet, and frequency of meeting.

The outcome indicators for the evaluation of the communications were mental health; K6, a 6-item screening scale for psychological distress [27]; subjective self-rated health [28]; and lifestyle habits, such as drinking and smoking. Moreover, this study investigated trust with the family [29]; subjective happiness level [30]; perceived social position, as assessed by a social stratification ladder called the Cantril Ladder [31]; and cohesion with neighborhoods [32]. These validated indicators were selected to assess the psychosocial health status.

### Statistical Analysis

First, a simple tabulation was presented about the basic attributes and separation status of the research participants. Furthermore, the communication tools were divided into 2 groups: “live,” which included voice and video options, such as phone call, internet free call, and video call; and “nonlive,” which included sending texts via email, group text chats such as LINE, and SMS text message. Communication frequency was calculated as the average score for each combination of the most frequently used tool and the next most frequently used tool. Participants with high-frequency scores, including both live and nonlive tools used as the first and second choices, were classified into the high communication–skilled (HCS) group and compared with the remaining participants, now treated as the reference group. For statistical comparison, the Student *t* test or the Wilcoxon rank-sum test was used for continuous variables depending on the observed data distributions. Similarly, the chi-square test or Fisher exact test was used for categorical variables. After making a simple comparison, the effects of HCS were estimated by adjusting the educational history, age, gender, and having a job or not, which were thought to be confounded by a multivariate regression model. For the statistical estimation of the effects of HCS on psychological health–related outcomes, logistic regression was used for dependent variables with a binary value of 0 or 1, and ordinary least-squares linear regression was used for continuous dependent variables. The significance level (*P* value) of all statistics was 5%, and analysis was performed using Stata version 16 (StataCorp, Inc.).

### Ethics Approval

The Kyushu University Medical District Institutional Review Board for Clinical Research approved the protocol of this verification study in 2018 (approval no. 30-335). The main study has also been approved for implementation.

## Results

Among the study participants who agreed to join the survey, 73 completed the questionnaire, including 61 family pairs consisting of couples or parent-child relationships. Their basic demographic characteristics and the status of separate living are summarized in Table 1. Mobility data on returning home to meet the family differed according to distance, and these were summarized based on the difference between domestic and international travels. A total of 53 pairs lived separately in the same country, 7 pairs were separated internationally, and 1 pair did not provide their residence status.

**Figure 1:**
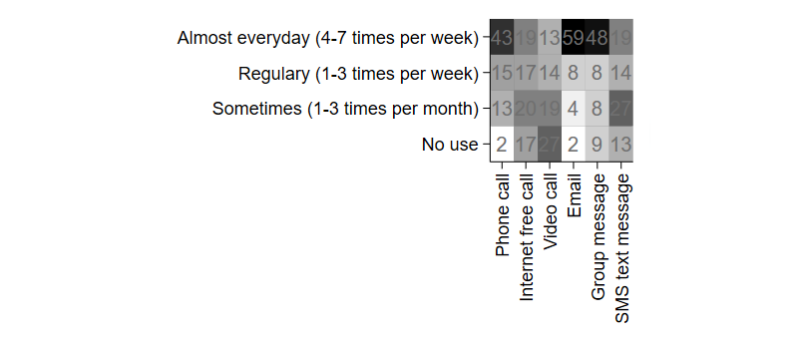
Types and frequencies of communication tools used between separated families (n=73; multiple choices for tool types).

Figure 1 shows the types of ICT tools used in family communication and the communication frequency among family members. ICT tools were divided into 2 types of systems: live and nonlive. Phone calls were the most frequently used option by study participants for family communication among the live-type systems. Among nonlive-type systems, most used email every day.

The study participants were summarized for each combination of the most frequently used and the second most frequently used tool for family communication. The average value of the scores of communication frequencies for each combination and the number of participants are shown in Table 2. The combination tools that had the highest average score for frequency of use of the combined tools (ie, both live and nonlive systems) were (1) phone calls and SMS text messages (score 16.3 + 6.4; n=9), video calls and group messages (15.5 + 4.6; n=6), or video calls and SMS text messages (score 14; n=1). The 15 people included in these combinations were chosen as the HCS group. We then compared the health and psychosocial states of the HCS group with the rest of the participants, who were considered the reference group.

Tables 3 and 4 list and compare the attributes of the HCS and reference groups. The HCS group included more graduates (15/16, 94%) than the reference group (29/57, 51%). However, there were no statistically significant differences in other attributes, such as age (*P*=.37), gender ratio (*P*=.94), engaged in a job (*P*=.58), or living status (*P*=.70). In addition, there was no difference in the status of separation between the HCS and reference groups on the frequency of actual meeting with the separated family, the travel time, and the cost.

A simple comparison was conducted with the reference group regarding health status, lifestyle, and evaluation of life and surroundings in the HCS group (Table 5). No significant differences were observed in health (psychological distress, *P*=.08; self-rated health, *P*=.07) or lifestyle (drinking, *P*>.99; current smoking, *P*=.37). The HCS group also showed no statistical difference in dyadic trust in family members living separately with that of the control group regarding interpersonal relationships (*P*=.80). However, the HCS group had higher scores of neighborhood social cohesion (*P*=.03), perceived higher social positions (*P*=.01), and higher level of happiness (*P*<.001) than the reference group.

**Table 1:** Basic characteristics of the study participants (n=73).

Characteristics				Value
Age (years), mean (SD)				45.5 (10.1)
Gender (female), n (%)				45 (62)
Education (graduate school), n (%)				44 (60)
Job (yes), n (%)				68 (93)
Living status (living alone), n (%)				53 (73)
Resident area (domestic), n (%)				69 (95)
**Familial relationship (n=61)^a^, n (%)**				
	Spouse			54 (89)
	Parent-child			7 (11)
Separation period, median (range)				1.7 years (1 month-12.7 years)
**Return home to meet the family**				
	**Once and more per month (yes), n (%)**			
		Domestic (n=54)	32 (59)	
		Foreign (n=7)	1 (14)	
	**Travel time (hours), median (25%-75%)**			
		Domestic (n=54)	4 (3-6)	
		Foreign (n=7)	15 (10-20)	
	**Travel fee (US $)^b^, median (25%-75%)**			
		Domestic (n=54)	136 (91-273)	
		Foreign (n=7)	909 (545-1182)	

^a^Not both of the paired family members are responding.

^b^Most of the respondents answered in yen, which was converted to US $ (US $1=¥110).

**Table 2:** Communication frequency score by the combination^a^ of the first and second most used tools in family^b^.

Communication tool	Phone call, n; mean (SD)	Free internet call, n; mean (SD)	Video call, n; mean (SD)	Email, n; mean (SD)	SMS text message, n; mean (SD)
Group message^c^	17; 10.2 (3.7)	11; 10.9 (3.0)	*6; 15.5 (4.6)*	4; 11.5 (3.7)	3; 16.0 (5.6)
SMS	*9; 16.3 (6.4)*	1; 6 (0)	*1; 14 (0)*	1; 10 (0)	N/A^d^
Email	6; 9.7 (3.0)	N/A	N/A	N/A	N/A
Video call	5; 18.4 (4.7)	4; 11.50 (6.14)	N/A	N/A	N/A
Free internet call	5; 12.4 (3.7)	N/A	N/A	N/A	N/A

^a^The combination tool including both “live” types (voice and video) and “nonlive” types (text).

^b^Italicized numbers in each cell indicate that the mean scores for communication frequency are relatively higher than others concerning the combination of both types of ICT tools.

^c^Group message means group-based text chat using Facebook messenger, Skype, and LINE, among others.

^d^N/A: not applicable.

**Table 3:** Comparison of the basic characteristics between the high communication skilled group and the reference group.

Characteristics	High communication skilled (n=16)	Reference (n=57)	*P* value
Age (years), mean (SD)	47.7 (10.1)	44.9 (11.2)	.37^a^
Gender (female), n (%)	10 (63)	35 (61)	.94^b^
Education (graduate school), n (%)	15 (94)	29 (51)	.002^c^
Job (yes), n (%)	16 (100)	52 (91)	.58^c^
Living status (living alone), n (%)	5 (31)	15 (26)	.70^b^
Resident area (domestic), n (%)	15 (94)	54 (95)	>.99^c^

^a^*P* value was calculated using the Student *t* test for continuous variables assumed to be a normal distribution.

^b^*P* value was calculated using the chi-square test for categorical variables; the sample is large enough (>5 [observed number in the sample] in matrix cell).

^c^*P* value was calculated using the Fisher exact test for categorical variables; the sample was not large enough (<5 [observed number in the sample] in any matrix cell).

**Table 4:** Comparison of the characteristics between the pairs in the high communication skilled group and the pairs in the reference group.

Characteristics				High communication skilled pairs (n=13)	Reference pairs (n=48)	*P* value
**Familial relationship**						.95^a^
	Spouse, n (%)			13 (100)	41 (85)	
	Parent-child, n (%)			0 (0)	7 (15)	
Separation period (years), median (range)				1.7 (0.7-3.7)	1.7 (1.3-2.7)	.33^b^
**Return home to meet family**						
		**Once and more per month (“Yes”), n/N (%)**				
			Domestic (n=54)	7/12 (55)	25/42 (60)	.94^c^
			Foreign (n=7)	1/1 (100)	0/6 (0)	.14^b^
		**Travel time (hours), median (25%**-**75%)**				
			Domestic (n=54)	4 (3.3-5.5)	4.5 (3-6)	.93^a^
			Foreign (n=7)	9 (—)	15.5 (12-20)	.29^a^
		**Travel fee^d^, median (25%-75%)**				
			Domestic (n=54)	159 (136-250)	136 (73-273)	.24
			Foreign (n=7)	1091 (—)	773 (545-1182)	.86

^a^*P* value calculated using the Wilcoxon rank-sum test for continuous variables assumed not to be a normal distribution.

^b^*P* value was calculated using the Fisher exact test for categorical variables; the sample was not large enough (<5 [observed number in the sample] in matrix cell).

^c^*P* value was calculated using the chi-square test for categorical variables; the sample is large enough (>5 [observed number in the sample] in matrix cell).

^d^Most respondents answered in yen, which was converted to US $ (US $1=¥110).

**Table 5:** Simple comparison of health, lifestyle, and psychological evaluation with human relationships and life between the high communication skilled and reference groups.

Comparison items of health, lifestyle, and psychological evaluation to human relationships	High communication skilled (n=16)	Reference (n=57)	*P* value^a^
Psychological distress (“Bad” by K6^b^), n (%)	2 (13)	21 (37)	.08
Self-rated health (“Bad” or “Not good”), n (%)	2 (13)	22 (39)	.07
Drinking (“≥20 g alcohol/day”), n (%)	3 (19)	10 (18)	>.99
Current smoking (“Yes”), n (%)	1 (6)	8 (14)	.37
Dyadic trust (scores), median (25%-75%)	48 (40-55)	48 (40-51)	.80
Neighborhood social cohesion (scores), median (25%-75%)	16 (16-20)	16 (14-17)	*.03* ^c^
Perceived social position (points), median (25%-75%)	7 (7-8)	7 (5-7)	*.01* ^c^
Life satisfaction (points) median (25%-75%)	8 (8-9)	7 (6-7)	*<.001* ^c^

^a^*P* values by Wilcoxon rank-sum test for continuous variables and Fisher exact test for categorical variables.

^b^“Bad” is defined by a cutoff point of 5 and more scores by the K6 to screen for psychological distress.

^c^Statistically significant values are in italics.

One’s level of education, communication methods, and evaluation of life may be influenced by educational history. Therefore, a multivariate regression analysis adjusted for confounding factors, such as educational history, age, gender, and job status, was performed (Tables 6 and 7). As a result, poor subjective health showed a prevalence odds ratio of less than 1 (0.17, 95% CI 0.03**-**1.02), but psychological distress (*P*=.09), lifestyle (drinking, *P*=.60; current smoking, *P*=.36), and dyadic trust with a family partner (*P*=.93) showed no significant association with those of the reference group, as was the case with the simple comparison. In the HCS group, the cohesion score with the neighborhood (β=2.40, 95% CI 0.56**-**4.24), perceived social position (β=1.17, 95% CI 0.11**-**2.23), and happiness level (β=1.46, 95% CI 0.58**-**2.34) were all higher.

**Table 6:** Results of multivariate regression of the association of health and lifestyles with the high communication–skilled group who used various types of information and communications technology tools for higher frequent communication with family.

Items of health and lifestyle			Prevalence odds ratio (95% CI)		*P* value		Adjusted^a^ prevalence odds ratio (95% CI)		*P* value
**Psychological distress (“Bad” by K6^b^)**									
	Reference	1				1			
	High communication skilled	0.24 (0.05-1.18)		.08		0.23 (0.04-1.23)		.09	
**Self-rated health (“Bad” or “Not good”)**									
	Reference	1				1			
	High communication skilled	0.23 (0.05-1.10)		.07		0.17 (0.03-1.02)		.05	
**Drinking (“≥20 g alcohol/day”)**									
	Reference	1				1			
	High communication skilled	1.08 (0.26-4.53)		.91		1.59 (0.29-8.69)		.60	
**Current smoking (“Yes”)**									
	Reference	1				1			
	High communication skilled	0.41 (0.05-3.53)		.42		0.34 (0.03-3.49)		.36	

^a^Adjusted for level of education, age, gender, and employment.

^b^“Bad” is defined by a cutoff point of 5 and more scores by the K6 to screen for psychological distress.

**Table 7:** Results of multivariate regression of the association of psychological status with the HCS group that used various types of ICT tools for higher frequent communication with family.

Items of psychological status			Correlation coefficient (β) (95% CI)		*P* value		Adjusted^b^ β (95% CI)		*P* value
**Dyadic trust (scores)**									
	Reference	0				0			
	High communication skilled	−0.02 (−4.95 to –4.90)		.99		0.24 (−4.28 to 5.6)		.93	
**Neighborhood social cohesion (scores)**									
	Reference	*0*				*0*			
	High communication skilled	*2.04 (0.37 to 3.71)*		*.02*		*2.40 (0.56 to 4.24)*		*.01*	
**Perceived social position (points)**									
	Reference	*0*				*0*			
	High communication skilled	*1.14 (0.17 to 2.09)*		*.02*		*1.17 (0.11 to 2.23)*		*.03*	
**Life satisfaction (points)**									
	Reference	*0*				*0*			
	High communication skilled	*1.40 (0.60 to 2.19)*		*.001*		*1.46 (0.58 to 2.34)*		*.002*	

^a^Italics indicates statistical significance for estimated values by regression analyses.

^b^Adjusted for level of education, age, gender, and employment.

## Discussion

### Principal Findings

In this study, people who frequently communicated using ICT took more advantage of its characteristics even if they were separated from their families, experienced more social cohesion with their neighborhood, and exhibited a higher degree of social position and a higher level of happiness in their life than people who did not communicate frequently using various ICTs. There was no relationship between the variety and frequency of communication methods and the sense of trust in the other family member living separately. In addition, as long as they communicate with each other with sufficient frequency by taking advantage of ICTs, separated persons from family maintained good health, although it was not statistically significant.

Separation, such as a single job transfer process without family, causes a great inconvenience in life, making the health condition worse. Therefore, single job-transferred workers have reported worse health than workers who move with family at job transfer [10,11]. However, this study could modify these findings. Our results show that a separated person who communicates more using various ICT tools may have a useful skill in maintaining good mental health. The study design was different from previous studies, and people who were not separated were not evaluated as a reference group in this study. However, good psychosocial effects were achieved by taking advantage of ICT tools and having enough frequency of communication, which can be useful for mitigating the inconvenience and stress in the life of single job-transferred workers in the future. Preferably, to identify the effective intervention methods necessary to improve the quality of life of separated families, additional studies are needed to evaluate the relationship between the state of detailed communication and the sociopsychological health status of both individuals separated and not separated.

It would be meaningful to evaluate the psychological health status of separated families considering their current infrastructure background, as ICT tools are now highly developed and the mobility of traffic movement has become increasingly flexible. In Japan, family members often separate temporarily for job reasons, and single job transfers were introduced by many Japanese companies during the period of high economic growth starting in the 1960s and 1970s and have been widely accepted among families. According to Japan’s public employment statistics, a survey on the number of households having a worker of single job transfer began in 1981. Since then, several evaluations have been conducted, focusing on the stress status of family members, the roles of parents, and the development of children [8]. Recent studies have also revealed that single-living workers separated from their families have poor lifestyles, poor psychological conditions, and poor medical examination results [10,11]. However, new communication devices, such as smartphones and tablets, are expected to significantly enhance the effects of communications using the internet, which were introduced after 2009 and have developed continuously. When using a smartphone, there were a few technical difficulties, such as the need for other devices and systems, at the time of its introduction. The communication cost is relatively low because the existing internet network can be used. In addition to texts, it is possible to communicate with a large amount of information, such as images and videos, so it has become possible to evaluate psychological health conditions considering the relationship of health conditions with both quality and quantity of communication methods. In Japan, the ownership rate of different information and telecommunications devices indicated that smartphones were the most common, accounting for up to 83.4% in 2019 [14].

Previous studies have already pointed out that the psychological state of single job-transferred workers may be improved by improving communication. It has been observed that when single-living employees have a health problem, more than half of them consult with a family member living far away rather than with colleagues or medical staff closer to them [10]. Thus, there is a possibility that sociopsychological indicators can be kept high by mastering and using diversified means of communication. As ICT is expected to continue to develop in the future, our results suggest evaluating the current transitional situation. In any case, as long as people learn and use ICT tools appropriately, the range of communication means will expand, eventually giving more merit.

### Limitations

As this study used a cross-sectional design, causal relationship cannot be proven. The relationship between cause and effect was not evaluated, such as whether the sociopsychological utility, such as happiness level, has increased due to sufficient communication, or whether the communication has become active because separated individuals had an insufficient connection with the surroundings and a suitable level of perceived social position. A longitudinal research design in the near future can be adopted to monitor life evaluation changes and the physical and mental health status before and after the start of separation or during separation and after the end of the separation period together with the quality and quantity of communication.

In addition, because only few participants (n=73) were evaluated in this study, there is a possibility that participants’ characteristics are biased. Looking at the attributes, it is possible that a large number of highly educated individuals with high socioeconomic status have gathered, so caution should be exercised when generalizing the results. Comparing single job-transferred employees with those living with family members, many single job-transferred employees held executive and managerial posts [11]. The annual incomes of these single job-transferred employees were usually higher than the average annual income of Japanese employees [8]. Therefore, participants in this study possibly had higher education and socioeconomic status than general people in households, including no single job-transferred members. In other words, highly educated people were likely to have family members living separately, so the results may apply to many households whose members live separately.

Moreover, this study used subjective health evaluation and did not use objective indicators, such as health checkup measurements, as in past studies. No statistically significant difference in the health indicators considered in this study might be attributed to the small number of participants, and the power was low. As we did not extract households, including single job-transferred workers, from a specific company or group, the diversity of evaluation targets was one of the strengths of the study when generalizing the findings. However, the small number of study participants threatened the reliability of research evaluation.

### Conclusions

Despite the aforesaid research limitations, those who frequently communicate with separated family members by taking advantage of various ICT tools can maintain a better mental state and better social relations among those who live alone and are separated from their families. This study suggested that cohesion with the surroundings, subjective social position, and happiness level are higher among those who communicate better using various tools and more frequently than those who have less communication. It is expected that there will be increasing opportunities to go to a new location, workplace, or school alone away from the family in this contemporary society according to ICT development and the increasing frequency of mobility. Therefore, it would be useful to evaluate how to communicate better to maintain a good mental state using technical aspects and frequency indicators in future studies.

## Appendix

Multimedia Appendix 1 Questionnaire for the ICT usage status.

## Abbreviations

HCS: high communication skilled
ICT: information and communications technology
SNS: social networking service
