# Supplementary material for: Communications Through Contemporary Tools of Information and Communication Technology: Cross-sectional Study Evaluating Health Among Separated Family Members
Source: JMIR Form Res. 2022 Aug 3;6(8):e34949. doi: 10.2196/34949 (PMC9386581; doi:10.2196/34949)
Supplement: Multimedia Appendix 1 [file formative_v6i8e34949_app1.docx]

Usage status and frequency of use of communication tools

We would like to ask you about the usage of your "usual" communication tool and the frequency of communication with your "remote family".

1 Do you have a cell phone? Please choose **only one** of the following:

Yes

No

2 Do you have a contract with a cell phone or a land phone such as "unlimited calls"? Please choose **only one** of the following:

Yes

No

Don't know

("Unlimited calls" means the service of unlimited talking with a fixed call rate.)

3 To what extent do you usually use the following communication tools for both work and private requirements? "Do not use" ~ "Use almost everyday," please select the most applicable frequency of use for each tool.

*Please choose the appropriate response for each item:

|  | **Do not use** | **One day per month** | **Couple of days per month** | **One day per week** | **2-3 days per week** | **4-5 days per week** | **Almosteveryday** |
| --- | --- | --- | --- | --- | --- | --- | --- |
| **Voice call (land phone, mobile phone)** |  |  |  |  |  |  |  |
| **OnLine voice free calls (LINE, Skype etc.)** |  |  |  |  |  |  |  |
| **Video call (Skype, Facetime etc)** |  |  |  |  |  |  |  |
| **SMS: Short Message Service / text message (text transmission / reception service to be sent to a telephone number between mobile phones and PHSs)** |  |  |  |  |  |  |  |
| **Group messages such as Line or FB messenger (through an account or email address on the Internet)** |  |  |  |  |  |  |  |
| **Electronic mail** |  |  |  |  |  |  |  |

4 To what extent do you use each communication tool with **your family member living separately in a remote area ("remote family")**? Please select the answer that applies to your situation.

*Please choose the appropriate response for each item:

|  | **Do not use** | **Use but less than**  **mentioned above** | **Use as above** |
| --- | --- | --- | --- |
| **Voice call (land phone, mobile phone)** |  |  |  |
| **OnLine free voice calls (LINE, Skype etc.)** |  |  |  |
| **Video call (Skype, Facetime etc.)** |  |  |  |
| **SMS: Short Message Service / text message (text transmission / reception service to be sent to a telephone number between mobile phones and PHSs)** |  |  |  |
| **Group messages such as Line or FB messenger (through an account or email**  **address on the Internet)** |  |  |  |
| **Electronic mail** |  |  |  |

5 Please select the two most frequently used communication methods (the first & the second) that you use with your remote family.

* All your answers must be different and you must rank in order. Please select at most 2 answers. Please number each box in order of preference from 1 to 6. Please choose no more than 2 items.

Voice call (land phone, mobile phone)

On-Line free voice calls (LINE, Skype etc.)

Video call (Skype , Facetime etc.)

SMS: Short message service / text message (text transmission / reception service to be sent to a telephone number between mobile phones and PHSs)

Group messages such as Line or FB messenger (through an account or email address on the Internet)

Electronic mail
